# The complete mitochondrial genome of the edible and phytopathogenic fungus *Desarmillaria tabescens*

**DOI:** 10.1080/23802359.2018.1535861

**Published:** 2018-11-25

**Authors:** Hwa-Yong Lee, Suyun Moon, Chang-Duck Koo, Jong-Wook Chung, Hojin Ryu

**Affiliations:** a Department of Biology, Chungbuk National University, Cheongju, Republic of Korea;; b Department of Forest Science, Chungbuk National University, Cheongju, Republic of Korea;; c Department of Industrial Plant Science and Technology, Chungbuk National University, Cheongju, Republic of Korea

**Keywords:** *Desarmillaria tabescens*, mitogenome, mushroom

## Abstract

*Desarmillaria tabescens* is one of the most important edible, medicinal, and phytopathogenic basidiomycetes. The complete mitochondrial genome of this species was determined using next-generation sequencing technology. This mitogenome is a circular molecule of 93,439 bp with a GC content of 29.28% and contains 15 protein-coding, two rRNA (*rnl* and *rns*), and 24 tRNA genes. Phylogenetic analysis revealed that *D. tabescens* is genetically closest to *Agrocybe aegerita*. *Desarmillaria tabescens* mitogenome can contribute to our understanding of the phylogeny and evolution of this species.

*Desarmillaria tabescens* was classified in the genus *Armillaria* but has been recently reassigned to genus *Desarmillaria* based on phylogenetic analysis of 6 genetic loci (28S nuclear ribosomal large subunit DNA, *Elongation Factor 1-α* [*EF1α*], *RNA polymerase II* [*RPB2*], *Actin-1* [*actin-1*], *Glyceraldehyde-3-Phosphate Dehydrogenase* [*gpd*], and *Beta-Tubulin* [*TUB*] and phenotypes such as exannulate mushroom formation and rhizomorph development under natural conditions (Koch et al. 2017). *Desarmillaria tabescens* is an edible mushroom (Sterry and Hughes 2009) and is considered a potent hepatoprotective remedy (Lu et al. 2007). Under natural conditions, this mushroom causes armillaria root rot (Amiri et al. 2008; Baumgartner et al. 2018) and participates in a symbiotic relationship with *Galeola septentrionalis* (Terashita and Chyuman 1987). Studies related to *D*. *tabescens* had been primarily limited to its phylogenetic relationships and distribution (Hasegawa et al. 2011; Coetzee et al. 2015; Park et al. 2017) and phytopathology (Amiri et al. 2008; Baumgartner et al. 2018). This study is the first to report the complete mitogenome sequence of *D*. *tabescens* (GenBank accession no. MH823225) and the phylogenetic analysis of this mushroom in relation to other species.

The A014 strain of *D*. *tabescens* used in this study was isolated from a fruit body of *Quercus acutissima* collected at Chungbuk National University (Cheongju, Chungcheongbuk-do, Korea; N36°37′43.64″, E127°27′04.87″). The strain has been deposited and maintained at the Korean Agricultural Culture Collection (Wanju, Jeollabuk-do, Korea) under the accession number KACC 54704. Total genomic DNA was extracted from mycelia cultured in malt extract broth media for 10 days under dark conditions using a GenEX Plant Kit (Geneall, Seoul, Korea) following the manufacturer’s instructions and sequenced using an Illumina Miseq Platform. Illumina paired-end reads were implemented *de novo* assembly after filtered the high-quality reads (>20 phred) using CLC *de novo* assembler (version 4.2.1, https://www.qiagenbioinformatics.com/products/clc-assembly-cell/; Kim et al. 2015). In brief, the mitochondrial contigs were compared with *Flammulina velutipes* mitochondrion sequence (GenBank accession JF799107) as reference by NUCmer tool in MUMmer package. Then merger of selected mitochondrial contigs were by read mapping using CLC read mapper. The genes in the mitogenome were annotated using GeSeq (https://chlorobox.mpimp-golm.mpg.de/geseq-app.html; Tillich et al. 2017), and manual confirmed using Artemis annotation tool was based on BLAST searches.

The complete mitogenome of strain A014 of *D*. *tabescens* is a circular molecule of 93,439 bp with a GC content of 29.28%. This mitogenome contains 15 protein-coding, 24 tRNA, and two rRNA (*rnl* and *rns*) genes. The 15 conserved protein-coding genes include seven subunits of NADH dehydrogenase (*nad1*, *nad2*, *nad3*, *nad4*, *nad4L*, *nad5*, and *nad6*), thee subunits of cytochrome c oxidase (*cox1*, *cox2*, and *cox3*), three subunits of ATPase (*atp6*, *atp8,* and *atp9*), apocytochrome b (*cob*), and ribosomal protein S3 (*rps3*). The 24 tRNA genes cover all 20 standard amino acids and range in size from 71 to 84 bp.

Phylogenetic analysis was conducted for the mitogenome and 10 other mushroom species using the neighbour-joining method of MEGA 7.0 with 1000 bootstrap replicates (Kumar et al. 2016). The phylogenetic tree indicated that the mitogenome of this species was genetically closest to that of *Agrocybe aegerita* (Figure 1). The mitogenome of *D. tabescens* can contribute to our understanding of the phylogeny and evolution of this recently reassigned species.

**Figure 1.**
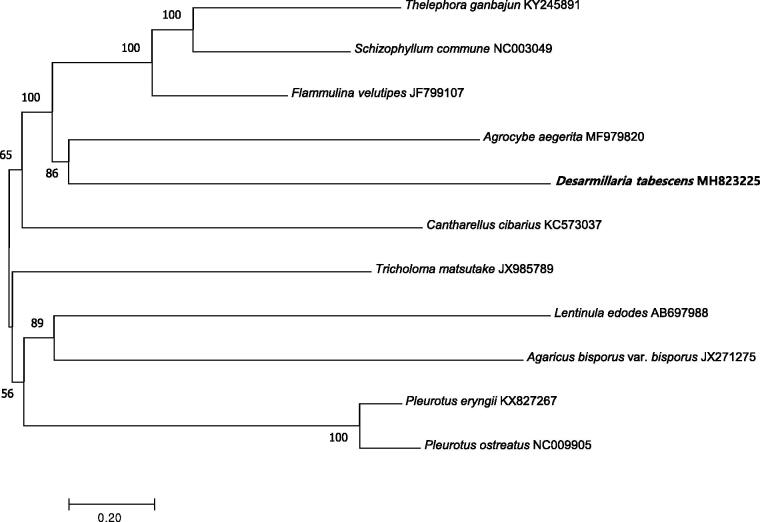
Neighbour-joining phylogenetic tree based on the mitochondrial genome sequences of *D*. *tabescens* and other 10 mushroom species using MEGA 7.0 (Kumar et al. 2016).
